# *COMT* Val^158^Met Polymorphism Modulates Huntington's Disease Progression

**DOI:** 10.1371/journal.pone.0161106

**Published:** 2016-09-22

**Authors:** Ruth de Diego-Balaguer, Catherine Schramm, Isabelle Rebeix, Emmanuel Dupoux, Alexandra Durr, Alexis Brice, Perrine Charles, Laurent Cleret de Langavant, Katia Youssov, Christophe Verny, Vincent Damotte, Jean-Philippe Azulay, Cyril Goizet, Clémence Simonin, Christine Tranchant, Patrick Maison, Amandine Rialland, David Schmitz, Charlotte Jacquemot, Bertrand Fontaine, Anne-Catherine Bachoud-Lévi, the French Speaking Huntington Group

**Affiliations:** 1 INSERM U955, Equipe 01 Neuropsychologie Interventionnelle, 94000, Créteil, France; 2 Département d’Etudes Cognitives, Ecole Normale Supérieure, PSL Research University, 75005, Paris, France; 3 Université Paris Est, Faculté de Médecine, 94000, Créteil, France; 4 ICREA, 08010, Barcelona, Spain; 5 Universitat de Barcelona, Departament de Cognició, Desenvolupament i Psicologia de L’Educació, 08035, Barcelona, Spain; 6 IDIBELL, Unitat de Cognició i Plasticitat Cerebral, 08907, L’Hospitalet de Llobregat, Spain; 7 Institut de Neurociència, Universitat de Barcelona, Barcelona, Spain; 8 INSERM-UPMC-CNRS, UMR 7225–1127, Institut Cerveau Moelle-ICM, Hôpital Pitié-Salpêtrière, 74013, Paris, France; 9 Assistance Publique-Hôpitaux de Paris, Département des Maladies du Système Nerveux, Hôpital Pitié-Salpêtrière, 74013, Paris, France; 10 Laboratoire de Sciences Cognitives et Psycholinguistique, ENS-EHESS-CNRS, Paris, 75005, France; 11 Assistance Publique-Hôpitaux de Paris, Département de Génétique, Hôpital Pitié-Salpêtrière, 74013, Paris, France; 12 Assistance Publique-Hôpitaux de Paris, Centre de Référence Maladie de Huntington, Service de Neurologie, Hôpital Henri Mondor-Albert Chenevier, 94000, Créteil, France; 13 CHU d'Angers, Centre de Référence des Maladies Neurogénétiques, Service de Neurologie, 49933, Angers, France; 14 CHU de Marseille—Hôpital de la Timone, Service de Neurologie et Pathologie du Mouvement, 13385, Marseille, France; 15 CHU de Bordeaux-GH Sud—Hôpital Haut-Lévêque, Service de Neurologie, 33604, Pessac, France; 16 CHRU de Lille, Service de Neurologie et Pathologie du Mouvement, 59000, Lille, France; 17 INSERM UMR-S 1172, JPArc, centre de recherche Jean-Pierre-Aubert neurosciences et cancer, Université de Lille, 59000, Lille, France; 18 CHU de Strasbourg—Hôpital de Hautepierre, Service de Neurologie, 67098, Strasbourg, France; 19 Assistance Publique-Hôpitaux de Paris, Hôpital Henri Mondor, Unité de Recherche Clinique, 94000, Créteil, France; Centre de Recherche Jean-Pierre Aubert, FRANCE

## Abstract

Little is known about the genetic factors modulating the progression of Huntington’s disease (HD). Dopamine levels are affected in HD and modulate executive functions, the main cognitive disorder of HD. We investigated whether the Val^158^Met polymorphism of the *catechol-O-methyltransferase* (*COMT*) gene, which influences dopamine (DA) degradation, affects clinical progression in HD. We carried out a prospective longitudinal multicenter study from 1994 to 2011, on 438 HD gene carriers at different stages of the disease (34 pre-manifest; 172 stage 1; 130 stage 2; 80 stage 3; 17 stage 4; and 5 stage 5), according to Total Functional Capacity (TFC) score. We used the Unified Huntington’s Disease Rating Scale to evaluate motor, cognitive, behavioral and functional decline. We genotyped participants for *COMT* polymorphism (107 Met-homozygous, 114 Val-homozygous and 217 heterozygous). 367 controls of similar ancestry were also genotyped. We compared clinical progression, on each domain, between groups of *COMT* polymorphisms, using latent-class mixed models accounting for disease duration and number of CAG (cytosine adenine guanine) repeats. We show that HD gene carriers with fewer CAG repeats and with the Val allele in *COMT* polymorphism displayed slower cognitive decline. The rate of cognitive decline was greater for Met/Met homozygotes, which displayed a better maintenance of cognitive capacity in earlier stages of the disease, but had a worse performance than Val allele carriers later on. *COMT* polymorphism did not significantly impact functional and behavioral performance. Since *COMT* polymorphism influences progression in HD, it could be used for stratification in future clinical trials. Moreover, DA treatments based on the specific *COMT* polymorphism and adapted according to disease duration could potentially slow HD progression.

## Introduction

Huntington’s disease (HD) is an autosomal dominant inherited neurodegenerative disease caused by increased number of CAG (cytosine adenine guanine) repeats in the Huntingtin (*Htt*) gene on chromosome 4 [1]. It primarily affects the striatum and manifests as progressive motor, behavioral and cognitive disturbances, leading to death about 15 to 20 years after onset. There is currently no effective course-modifying treatment.

Phenotypic expression differs considerably between patients. Age at onset varies and few of the underlying genetic factors for this variability have been identified [2]. The size of the number of CAG repeats in the mutated *Htt* (*mHtt*) gene is inversely related to age at onset of HD patients, but accounts for only 40 to 70% of its variance [3]. The implication of other genes in HD such as the *PPARGC1A*, *GRIK2*, *APOE* and *BDNF* genes, has been shown, but their impact was not replicated in subsequent studies [4, 5, 6]. The factors influencing disease progression remain to be identified [7]. Higher number of CAG repeats in the *mHtt* gene is associated with faster motor, cognitive, and functional decline [8]. The influence of the number of CAG repeats in the normal *Htt* allele remains uncertain, either on age at onset or disease progression [3, 9].

Here, in addition to results provided by genome wide association mapping conducted on the motor onset [10], we conduct an *a priori* study on the *catechol-O-methyltransferase* (*COMT*) to assess its impact on HD evolution [11]. COMT may play a role in HD because it degrades catecholamines, such as dopamine (DA). Medium-sized striatal spiny GABAergic neurons bearing dopaminergic receptors (D1 and D2) are preferentially affected in HD [12]. The density of these receptors in the striatum decreases [13] along with DA and GABA concentrations in HD patients. DA receptors loss correlates with disease progression in mouse HD models [14]. Furthermore, DA receptors dysfunction is correlated with cognitive impairment in HD gene carriers [15]. In the normal population, the presence of a Valine instead of a Methionine in position 158 (Val^158^Met) on the *COMT* gene on chromosome 22 increases *COMT* activity to levels 38% higher for the Val/Val genotype than for the Met/Met genotype [16], resulting in lower DA levels in Val/Val homozygotes. *COMT* polymorphism essentially affects DA levels in the prefrontal cortex (PFC), whereas striatal DA level is regulated principally by the DA transporter (DAT). However, there is an interaction between *DAT* and *COMT* genes in the regulation of DA level in the fronto-striatal system [17]. Indeed, *COMT* polymorphism influences the severity of cognitive and behavioral symptoms in other diseases affecting subcortical DA regulation, such as Parkinson’s disease [18, 19] and schizophrenia [20], and is predictive of disease progression and psychosis in 22q11.2 deletion syndrome [21], another disease related to striatal dysfunction. Additionally, in early stages of Huntington’s disease the PFC function appears to have an important role in compensation of cognitive impairment [22].

In HD, *COMT* polymorphism has no influence on motor onset [4], but its effect in behavioral, cognitive and functional domains has not been investigated except in a very recent study. In a cross-sectional study of 121 HD patients, Vinther-Jensen et al [23] found that *COMT* and *MAOA* polymorphism were associated with behavioral symptoms or cognitive impairment, respectively. The link between polymorphisms in genes involved in the dopaminergic pathway and the behavioral and cognitive symptoms highlights the role of dopamine regulation in HD symptomatology. However, patients were not assessed longitudinally, and the impact of *COMT* polymorphism in disease progression remains unexplored.

The cognitive effects of *COMT* polymorphism in various diseases and in healthy populations have repeatedly been reported to be specific to executive functions (see [24, 25] for reviews), and executive function defects are the hallmark of cognitive dysfunction in HD. Furthermore, even at low doses, DA aggravates *mHtt* toxicity in striatal neuron cultures [26] and increases behavioral and motor deficits in YAC128 mice [27], a transgenic model of HD. Thus, *COMT* polymorphism may affect the progression of HD.

In this study, we investigated the impact of *COMT* polymorphism on HD progression on cognitive, motor, behavioral and functional decline, in a longitudinal long-term prospective study.

## Material and Methods

### Participants

We report a longitudinal prospective long-term study of 438 HD gene carriers from the Predictive Biomarkers for Huntington’s disease protocol (NCT01412125), which was approved by the ethics committee of Henri Mondor Hospital (Créteil, France) in accordance with EU and French bioethics laws. All HD gene carriers gave written informed consent. They were heterozygous for the *Htt* gene (> 36 CAG repeats in *mHtt*) and aware of their genetic status. They had no other neurological conditions or long-term experimental treatment (e.g. cell transplantation).

Data were collected from 1994 to 2011, at eight centers from the French Speaking Huntington’s Disease Group (Angers: 24%, Bordeaux: 7%, Créteil: 34%, Lille: 4%, Lyon: 1%, Marseille: 12%, Paris: 11%, Strasbourg: 7%), and centralized at the National Reference Centre for Huntington’s disease in Créteil.

Blood samples were centralized at the DNA bank of Pitié-Salpêtrière Hospital. The number of CAG repeats was routinely determined [28]. The rs4680 (*COMT* Val^158^Met) polymorphism was genotyped by PCR with appropriate primers [29]. We investigated the distribution of *COMT* genotypes in the general population, by genotyping 367 independent controls with similar ancestry with the same technique.

### Clinical assessment

HD gene carriers were followed up with the Unified Huntington’s Disease Rating Scale (UHDRS) [30], which combines motor, functional, behavioral and cognitive assessments. Motor domain was assessed using the Total Motor Score (TMS, range: 0 to 124). Functional domain was assessed using the Total Functional Capacity scale (TFC, range: 13 to 0), Functional Assessment Scale (FAS, range: 25 to 50) and Independence Scale (IS, range: 100 to 0). Behavioral domain was assessed using the psychiatric part of the UHDRS (range: 0 to 88). Cognitive domain was assessed using the Stroop Test (color naming: Stroop C, word reading: Stroop W, and color-word interference: Stroop C/W), Symbol Digit Modality Test (SDMT), and letter fluency (for P, R and V in French). For letter fluency, testing at two minutes appears to be more sensitive than testing at one minute [31]. The French version used in this study includes both measurements. Higher scores in IS, FAS and TMS indicate greater impairment. For all other tasks, higher scores indicate lower impairment.

The first evaluation corresponding to the entrance in the study (first visit) occurred before onset (pre-manifest) in some individuals and at various times after onset in others, such that the sample encompassed the entire spectrum of HD progression (first visit: 8% pre-manifest gene carriers; 39% patients at Stage 1; 30% Stage 2; 18% Stage 3; 4% stage 4; and 1% Stage 5). Pre-manifest gene carriers were defined as having a TMS below or equal to 5 [32], and a TFC score of 13. The visits were performed annually, with few exceptions, with a mean inter-visit delay of 1.2 years (SD = 0.4). The mean number of visits per HD gene carriers was 5.0 (SD = 3.2; range: 1 to 19 visits). Thirty-two HD gene carriers were seen only once. Data were recorded for 2185 visits. The mean duration of follow-up was 4.3 years for the whole cohort (SD = 3.0; range: 0 to 15.5 years), and 4.8 years (SD = 2.9) years when excluding patients assessed once.

The date at onset was available for 86.53% of the HD gene carriers. It corresponds to the appearance of the first symptoms, and it was determined (observed) by the clinician (93.14%), or, if missing, by the family (5.28%), or, if missing that as well, by the participant (1.58%).

### Statistical analyses

#### Demographics and characteristics of COMT polymorphism groups at the first visit

The χ^2^ goodness-of-fit test was computed to compare the distribution of *COMT* genotypes in the 438 HD gene carriers and in the control group. Stability of the genotype frequency was assessed through the Hardy-Weinberg test in controls.

We assessed whether baseline characteristics of HD gene carriers were similar in the different *COMT* polymorphism samples (Met/Met, Val/Val and Met/Val), by first assessing the differences between groups for each score of the UHDRS at the first visit. Demographic data and clinical characteristics of the sample (N = 438) at the first visit were compared between groups, with a Pearson’s χ^2^ tests for qualitative variables and a one-way ANOVA for quantitative variables. For variables with significant difference between groups, student’s *t*-tests (or Welch’s tests in cases of unequal variances) were performed with Bonferroni correction for multiple pairwise comparisons (see S1 Table for the same comparisons in the subgroup included in the longitudinal analysis).

#### Number of CAG repeats and age at onset

We first assessed the impact of the number of CAG repeats on age at onset in the 379 HD gene carriers with these available data. We used a linear regression model, with age at onset as the dependent variable and the number of CAG repeats as an independent variable. The R^2^ value provided by the model is an estimate of the proportion of the variability of the age at onset explained by the number CAG repeats.

We also calculated an expected age at onset according to the Langbehn et al. model [33], derived from the number of CAG repeats using the formula: *expected age = (21*.*54 + exp (9*.*556–0*.146*CAG)). We evaluated the agreement between this expected age at onset and the age at onset provided in our database by calculating the intraclass correlation coefficient (ICC), a measure for concordance. The ICC was obtained by a two-way mixed effect model [34].

#### Longitudinal analysis of disease progression

The longitudinal analysis was conducted on 1912 visits of the 350 HD gene carriers with at least two visits and for which the date of onset was known.

**Fig 1:**
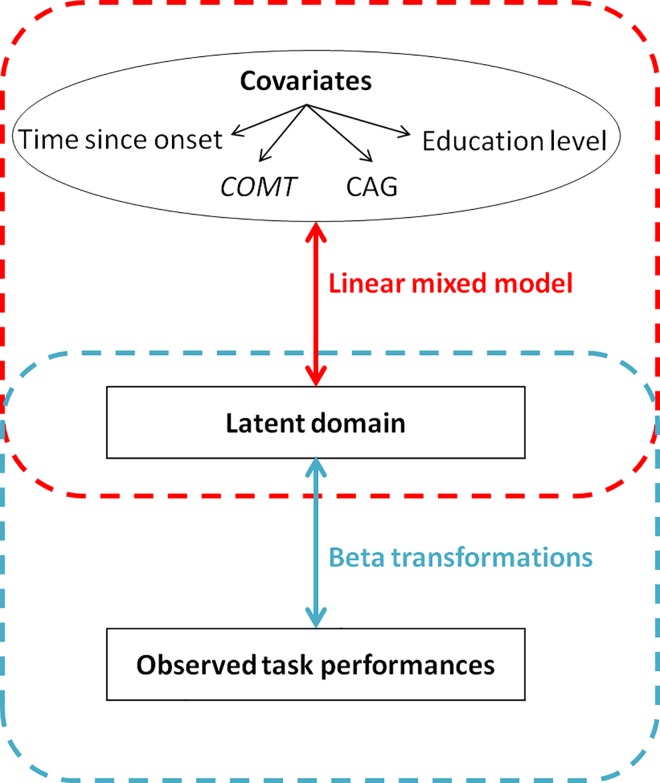
Structure of the latent class mixed models. Red dashed line includes variables used for the linear mixed model part. Blue dashed line includes variables used for the beta transformation. Latent domain represents the non-observable motor, behavioral, functional or cognitive domains. Observed task performances are those measured using the UHDRS. The latent motor process was modeled using the TMS; the latent behavioral process was modeled using the UHDRS behavioral score; the latent functional process was modeled using the FAS and IS scores; The latent cognitive process was modeled using letter fluency at 1 minute, letter fluency at 2 minutes, SDMT, Stroop Color, Stroop Word and Stroop Word/Color interference.

We compared progression over time between groups, by calculating the overall change in motor, functional, behavioral and cognitive domains per year since the date of onset. Domains are not observable *per se* but are modeled by a latent variable reflected by observed performances at each task. We performed four latent-class mixed models [35], one per domain, where each model combines: (i) a linear mixed model to explain latent domain according to covariates, and (ii) beta transformations which link observed performances at each task to latent domain (Fig 1). Similarly to classical linear mixed models, the latent-class mixed model allows integrating data from HD gene carriers with unequal duration of follow-up and introducing a subject-specific intercept by random effects to account for within-unit correlation for outcome and between-subject variability [36]. These models take into account all observations for each patient, without listwise deletion. Moreover, the use of beta transformations allows taking into account the ceiling and floor effects of UHDRS tasks.

Parameters of linear mixed model and beta transformations are estimated simultaneously using maximum likelihood method and Monte-Carlo integration. The disease duration (time since onset), *COMT* polymorphism, number of CAG repeats in *mHtt*, interaction between disease duration and the number of CAG repeats, interaction between disease duration and *COMT* polymorphism, gender [37] and education level were retained as covariates. For *COMT* polymorphism, included in the model as a categorical covariate, Met/Met was the reference group, allowing the comparison between Met/Met and Val/Val genotypes and between Met/Met and Met/Val genotypes. We compared Met/Val and Val/Val genotypes by recomputing the models with Met/Val genotype as the reference group. All *P*-values were adjusted with Bonferroni correction in two steps: one within the *COMT* polymorphism groups comparison and one for multiple comparisons across domains. Based on Akaike’s information criterion and Bayesian information criterion [38], number of CAG repeats in the normal *Htt* allele and CAG-*COMT* interaction did not improve model fit, thus they were removed from the final model. Similarly, accounting for the age at onset rather than for the number of CAG repeats did not improve model fit.

To assess the robustness of the results, a sensitivity analysis was performed excluding outliers, on the basis of the number of CAG repeats and of the distribution of dates of visits in our cohort (see S1 Fig and S2 Fig). The sensitivity analysis included HD gene carriers with a number of CAG repeats between 39 and 49 that were followed in the 20 years after disease onset.

Analyses were conducted with R 2.3 software (http://www.r-project.org/). The R package lcmm was used to perform the longitudinal analysis. All tests were two-tailed. Values of *P* < 0.05 were considered significant.

## Results

### Demographics and characteristics of COMT polymorphism groups at the first visit

The χ^2^ goodness-of-fit test confirms that the distribution of *COMT* genotypes is similar in HD gene carriers and the control group (*P* = 0.15) (see Table 1). The genotype frequencies remain constant from generation to generation (Hardy-Weinberg *P* = 0.13).

**Table 1:** Distribution of the *COMT* genotypes in HD gene carriers and control groups.

	Met/Met	Met/Val	Val/Val
Controls *N* (%)	70 (19.1)	202 (55.0)	95 (25.9)
HD gene carriers *N* (%)	107 (24.4)	217 (49.6)	114 (26.0)

HD: Huntington’s disease; Met: Methionine; Val: Valine

Demographic and clinical data of HD gene carriers for the first visit are displayed in Table 2. Baseline demographic and clinical characteristics are similar for all *COMT* polymorphisms (one-way ANOVA, *P* > 0.05) except that HD gene carriers with the Met/Val genotype have a lower educational level than those with the Val/Val (pairwise comparison, corrected *P* = 0.01) or Met/Met (pairwise comparison, corrected *P* = 0.01) genotypes. Descriptive analysis of HD gene carriers included in the longitudinal analysis is provided on S1 Table. *COMT* polymorphism does not impact age at onset.

**Table 2:** Demographic characteristics and performance of HD gene carriers.

	N	Met/Met	Met/Val	Val/Val	P*
Age (yrs)	438	46.1 (12.8)	49.5 (12.1)	47.9 (11.2)	Ns
Sex (% men)	438	55.1	47.0	52.6	Ns
Age at onset (yrs)	379	41.9 (11.6)	45.3 (11.5)	43.6 (9.7)	Ns
Educational level (yrs in education)	435	12.3 (3.4)	11.2 (2.9)	12.2 (3.3)	0.0012
BMI	382	22.6 (3.7)	22.7 (3.6)	22.1 (3.5)	Ns
CAG repeats *mHtt*	438	45.3 (4.5)	44.5 (3.6)	44.6 (3.1)	Ns
CAG repeats *Htt*	438	18.3 (2.8)	18.9 (4.1)	18.9 (3.9)	Ns
Antipsychotic use (%)	438	75.7	73.3	72.8	Ns
Antidepressant use (%)	438	28.0	27.6	28.1	Ns
Benzodiazepine use (%)	438	24.3	23.0	14.0	Ns
**UHDRS**					
TMS	421	30.6 (19.7)	32.3 (22.0)	35.9 (23.3)	Ns
Behavior	402	17.8 (13.4)	17.1 (11.0)	16.3 (12.0)	Ns
FAS	424	29.4 (5.3)	30.0 (5.9)	30.7 (6.0)	Ns
IS	425	84.3 (15.2)	83.0 (16.7)	81.1 (16.4)	Ns
TFC	438	9.4 (3.4)	9.3 (3.4)	8.8 (3.6)	Ns
L Fluency 1’	340	22.7 (12.9)	20.0 (12.6)	19.7 (13.1)	Ns
L Fluency 2’	350	33.3 (21.4)	28.8 (19.9)	28.2 (20.6)	Ns
Stroop W	364	64.4 (24.9)	61.9 (23.3)	65.4 (27.5)	Ns
Stroop C	365	47.8 (20.6)	43.8 (17.1)	46.7 (20.7)	Ns
Stroop W/C	362	25.8 (14.3)	23.6 (12.8)	23.6 (15.4)	Ns
SDMT	314	26.6 (16.6)	24.1 (15.2)	25.1 (17.3)	Ns

HD: Huntington’s disease; BMI: body mass index; CAG repeats refers to the number of CAGs in the mutated (*mHtt*) and non-mutated (normal *Htt*) alleles of the Huntingtin gene; UHDRS: Unified Huntington’s Disease Rating Scale; TMS: Total Motor Score; FAS: Functional Assessment Scale; IS: Independence Scale; TFC: Total Functional Capacity; Letter fluency (L Fluency) was tested with PRV letters (French norms) at 1 minute (1’) and 2 minutes (2’); Stroop C: Color; W: Word; W/C: Word/Color (interference score); SDMT: Symbol Digit Modalities Test. N: Number of patients for each variable where the data were available. Quantitative variables are presented as means, with the standard deviation in brackets, and qualitative variables are presented as frequency counts. Medication use is expressed as a percentage.

*Non corrected *P*-values; Chi-squared test for qualitative variables and one-way ANOVA for quantitative data; Ns: not significant.

#### Number of CAG repeats and age at onset

The number of CAG repeats explains 49.61% of the variability of age at onset (β coefficient = -2.07 (SE = 0.11), *P* < 0.001).

**Fig 2:**
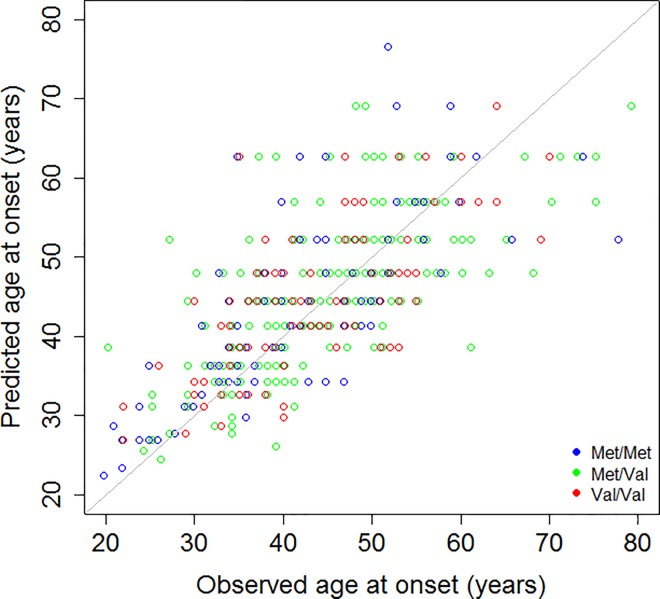
Concordance between predicted and real age at onset. Each point represents an individual patient. The observed age at onset is the one provided in the database. The predicted age at onset is the one calculated by the formula 21.54 + exp(9.556–0.146 x CAG). The gray line is the first bisector corresponding to the line of predicted = observed. The closeness of the points to the gray line indicates the extent to which predicted age at onset matches real age at onset. If predicted age at onset is greater than the observed age at onset, the points are located above the gray line. By contrast, if the predicted age at onset is below the real age at onset, the points are located below the gray line.

The ICC measuring agreement between expected age at onset by formula (1) and age at onset provided in the database is high for the whole cohort (0.71: [95% CI 0.65–0.76], *P* < 0.0001) and in each *COMT* group (Met/Met ICC = 0.75 [95% CI 0.64–0.83], *P* < 0.0001, Met/Val ICC = 0.70 [95% CI 0.62–0.77], *P* < 0.0001 and Val/Val ICC = 0.66 [95% CI: 0.54–0.76], *P* < 0.0001) (Fig 2).

### Longitudinal analysis of disease progression

**Fig 3:**
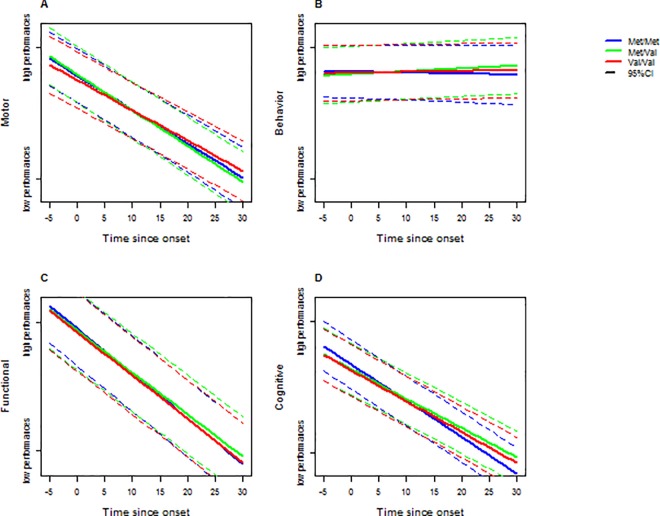
Curves of the impact of *COMT* polymorphism on the motor, behavioral, functional and cognitive domains, in a modeled cohort of a woman HD patients with 45 CAG repeats and 12-year education level. We plotted the evolution of performance as a function of time for each task. Performance decrease was represented by a negative slope. 45 CAG repeats is the mean number in the cohort studied. The latent motor process was modeled using the UHDRS motor score; the latent behavioral process was modeled using the UHDRS behavioral score; the latent functional process was modeled using the FAS and IS scores; The latent cognitive process was modeled using letter fluency at 1 minute, letter fluency at 2 minutes, SDMT, Stroop Color, Stroop Word and Stroop Word/Color interference.

**Fig 4:**
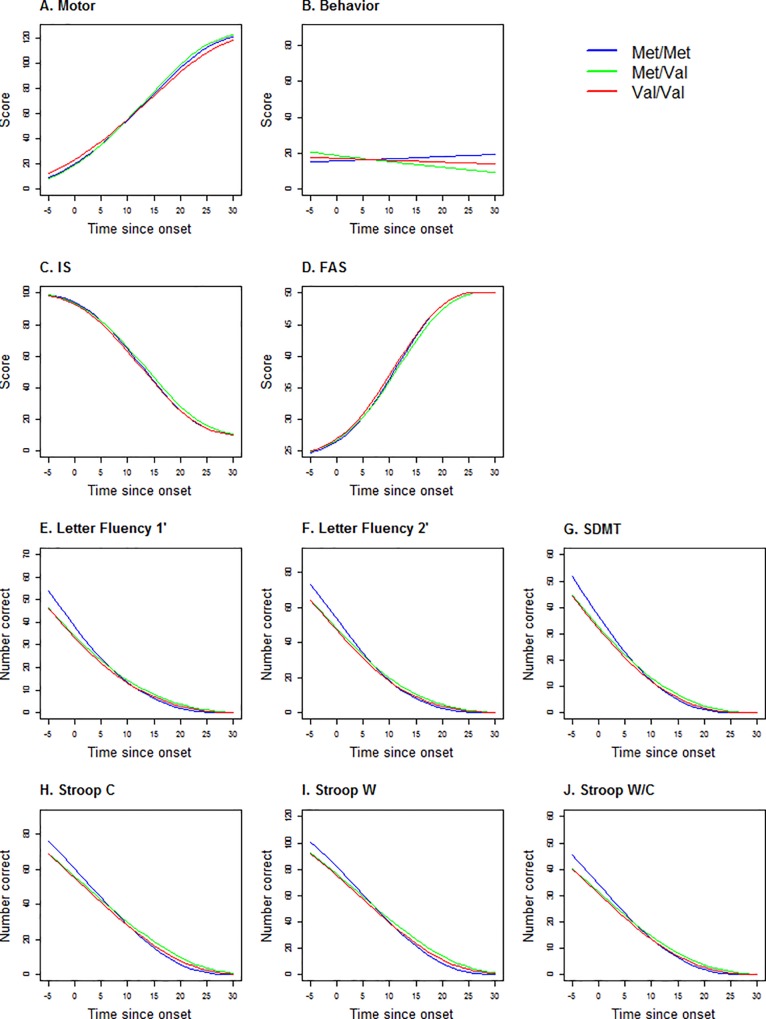
Curves of the impact of *COMT* polymorphism on each UHDRS score, in a modeled cohort of a female HD patient with 45 CAG repeats and 12-year education level. We plotted the evolution of performance for each task. 45 CAG repeats is the mean number in the cohort studied. UHDRS motor score **(A)**; UHDRS behavioral **(B)**, IS: Independence Score **(C)**; FAS: Functional Assessment Scale **(D)**, cognitive (letter fluency 1’: at 1 minute **(E)**; letter fluency 2’: at 2 minutes **(F)**; SDMT: symbol digit modalities test **(G)**; Stroop C: Stroop color **(H)**; Stroop W: Stroop word **(I)**; Stroop W/C: Stroop interference **(J**).

Table 3 displays the modeling parameters of the linear mixed models corresponding to the disease evolution within the four domains: motor, behavior, functional and cognitive. After correcting *P*-values, there is no effect of *COMT* polymorphism or the number of CAG repeats on latent processes at time 0 (estimated onset). A higher education level is correlated with higher performance in cognitive and functional domains. Gender influences the disease evolution. Men were behaviorally less impaired than women at onset. In addition, men decline more slowly than women in both the motor and cognitive domains. For all *COMT* polymorphism, performance declined over time for the motor, cognitive, and functional domains but not for behavior (see Fig 3). Higher number of CAG repeats is associated with a faster decline for motor, cognitive and functional domains. Met/Met HD gene carriers decline faster than Val/Val and Met/Val HD gene carriers in the cognitive domain. Met/Val HD gene carriers decline faster than Val/Val HD gene carriers in the motor domain while not differing from the Met/Met group. At age at onset and over the 10 years following disease onset, Met/Met HD gene carriers outperform Met/Val and Val/Val HD gene carriers in the cognitive domain. However, since they decline faster they subsequently perform less well than the other HD gene carriers (Fig 3 and Fig 4). The intersection of the progression curves for the Met/Met and Met/Val groups is estimated at 7.2 years for the cognitive domain. The intersection of the Met/Met and Val/Val curves is estimated at 10.9 years for the cognitive domain. The intersection of the Met/Val and Val/Val curves is estimated at 11.0 years for the motor domain.

**Table 3:** Impact of *COMT* genotype and the number of CAG repeats in the long allele on disease evolution within the four domains.

Domains	Motor	(N = 348)	Behavior	(N = 348)	Functional	(N = 348)	Cognitive	(N = 344)
	Estimate	*P*	Estimate	*P*	Estimate	*P*	Estimate	*P*
	(SE)	(Corrected *P*)	(SE)	(Corrected *P*)	(SE)	(Corrected *P*)	(SE)	(Corrected *P*)
**Baseline:**								
Met/Val vs Met/Met	0.08	0.6595	-0.39	0.1592	-0.10	0.5478	-0.30	0.0521
	(0.18)	(ns)	(0.28)	(ns)	(0.17)	(ns)	(0.16)	(ns)
Val/Val vs Met/Met	-0.26	0.1825	-0.18	0.5427	-0.19	0.3105	-0.37	0.0344*
	(0.20)	(ns)	(0.30)	(ns)	(0.19)	(ns)	(0.17)	(ns)
Val/Val vs Met/Val	-0.34	0.0383*	0.21	0.3708	-0.09	0.5654	-0.06	0.6605
	(0.16)	(ns)	(0.23)	(ns)	(0.16)	(ns)	(0.15)	(ns)
Number of CAG repeats	-0.01	0.4087	0.07	0.1202	0.04	0.0391*	0.03	0.0443*
	(0.02)	(ns)	(0.04)	(ns)	(0.02)	(ns)	(0.02)	(ns)
Education level	0.04	0.0500	0.05	0.0181*	0.06	0.0014**	0.08	<0.0001***
	(0.02)	(ns)	(0.02)	(ns)	(0.02)	(0.0056**)	(0.02)	(0.0001***)
Gender Man vs Woman	-0.04	0.7566	0.54	0.0082**	0.30	0.0233*	-0.04	0.7436
	(0.14)	(ns)	(0.20)	(0.0328*)	(0.13)	(ns)	(0.12)	(ns)
**Slope:**								
Met/Val vs Met/Met	-0.01	0.3671	0.06	0.0171*	0.02	0.1339	0.04	<0.0001***
	(0.01)	(ns)	(0.02)	(ns)	(0.01)	(ns)	(0.01)	(<0.0001***)
Val/Val vs Met/Met	0.02	0.1160	0.03	0.2961	0.01	0.4642	0.03	0.0002***
	(0.01)	(ns)	(0.03)	(ns)	(0.01)	(ns)	(0.01)	(0.0012**)
Val/Val vs Met/Val	0.03	0.0044**	-0.03	0.1711	-0.01	0.4700	-0.01	0.2221
	(0.01)	(0.0264*)	(0.02)	(ns)	(0.01)	(ns)	(0.01)	(ns)
Number of CAG repeats	-0.01	<0.0001***	-0.01	0.0953	-0.01	<0.0001***	-0.01	<0.0001***
	(0.001)	(<0.0001)	(0.004)	(ns)	(0.001)	(<0.0001***)	(0.001)	(<0.0001***)
Gender Man vs Woman	0.03	0.0016**	-0.06	0.0042**	-0.01	0.3862	0.02	0.0020**
	(0.01)	(0.0064**)	(0.02)	(0.0168*)	(0.01)	(ns)	(0.01)	(0.0080**)

The motor domain was modeled including the performances at TMS; the behavioral domain was modeled including the performances at behavior task of the UHDRS; the functional domain was modeled including the performances at FAS and IS (TFC could not be included because there are not enough values for the model to converge); the cognitive domain was modeled including performances at letter fluency assessed at 1 and 2 minutes, SDMT and the three parts of the Stroop.

N: Number of HD gene carriers who have contributed to the estimation (cognitive tasks were not available for all HD gene carriers); SE: Standard error of the estimate

*P*: *P*-values (*** P<0.001, ** P<0.01, *P<0.05).

*Baseline* values correspond to the impact of covariates at estimated age at onset. *Slope* values correspond to the impact of covariates on the slope of the decline.

Fig 4 shows that for most tasks, the fit of disease is linear only for the first 15 years, displaying a floor effect after that point. Beta link functions between performance at each task and latent variable modeling of the domains are displayed in S3 Fig.

In the sensitivity analysis, larger number of CAG repeats is associated with a faster decline over time, in all domains except behavior. Met/Val HD gene carriers decline faster than Val/Val HD gene carriers in motor domain. Met/Met HD gene carriers decline faster than Val/Val and Met/Val HD gene carriers in cognitive domain, but the associated *P*-value is no longer significant after Bonferroni correction (see S2 Table).

## Discussion

We investigated the impact of *COMT* polymorphism in a prospective multicentre study of 438 HD gene carriers at all stages of HD, from which 350, with identified age of onset, were followed up once a year with the UHDRS during 4.8 (SD = 2.9) years. The *COMT* polymorphism distribution in this sample is similar to that reported for the European population [39]. As previously reported, the number of CAG repeats affects the age at onset and the disease progression in our cohort [3, 9]. Higher educational level improves cognitive performance at baseline, as observed in elderly populations [40]. We show that *COMT* polymorphism influences disease progression in a biphasic way in the cognitive and motor domains, and tends to influence the functional domain as well. Met/Met HD gene carriers outperform Val/Val HD gene carriers in the cognitive during the first 10 years after disease onset. However, since the slope of decline is steeper in the Met/Met HD gene carriers, they then performed worse than Val/Val HD gene carriers. The effect of *COMT* polymorphism is of particular interest because, in contrast to other genetic modulations previously reported, this polymorphism affects progression rather than age at onset [2, 4].

This study replicates the effect of the number of CAG repeats observed in other studies [41, 42]. It allows deciphering the effect of *COMT* polymorphism presumably because unlike previous studies on other cohorts [43], we did not select HD gene carriers at particular disease stages or with a specific number of CAG repeats. In addition we improved the value of our results by selecting the number of CAG repeats without including the age at onset as a covariate despite its known value [8] to avoid redundancy [44] since the age at onset and the number of CAG repeats are two correlated factors [3, 33]. Furthermore, the use of a single language for cognitive testing decreased inter-subject variability in cognitive performance. The HD gene carriers were followed up prospectively for as long as possible, from pre-manifest to advanced stages. Although most of the data was collected between 5 and 15 years after disease onset, it provides a unique continuum of disease progression with enough follow up data to conduct a longitudinal analysis. Results of the sensitivity analysis displayed the same trend as the analysis of the whole sample, however without reaching significance after Bonferroni correction for multiple testing.

The latent-class mixed model has the advantage of grouping several tasks within domains and provides a global picture by domain without focusing on specific tasks. This approach, recently developed, is already used in studies evaluating cognitive decline [45, 46]. To ensure that it models disease progression as well as the classical task by task multiple linear mixed model [47, 48], we ran both latent-class mixed models and the linear mixed models on our data (see S3 Table). Both models show higher cognitive decline for the Met/Met group. The latent-mixed model has also the advantage of avoiding the calculation of sum of performance for tasks with different weights and to take into account all assessments, and not only a delta between baseline and last assessment, as in some regression linear analyses [49].

Our study shows that the impact of *COMT* polymorphism differs according to each domain like in previous studies of Parkinson’s disease and schizophrenia [19, 20, 50]. The effect of *COMT* polymorphism was observed in the cognitive and motor domains but not in the behavioral and functional domains. However, the pattern was similar in the motor, cognitive and functional domains, without reaching significance in the latter. The Val/Val group show a less steep progression than the Met/Met group in the cognitive domain, and than the Met/Val group in the motor domain. The lack of significance in the functional domain presumably relies on the smaller range of performance in this domain than in the motor and cognitive domains. It is worth noting that in contrast, the behavioral score does not show any decline overtime in HD.

The impact of *COMT* polymorphism on cognitive functions is of particular interest because the cognitive abilities are the major cause of social withdrawal in HD. Executive functions are modulated by *COMT* polymorphism, and improve along with the increase of DA availability, as observed in healthy individuals with the Met/Met genotype [51]. DA in the striatum has phasic variations and affects executive tasks requiring cognitive flexibility and switching between object features, such as in the Wisconsin Card Sorting Test and attention set-shifting test [52]. In the PFC, DA availability is more tonic and modulates the ability to maintain information as required in selective attention or working memory [53, 54]. The tasks we used here fall into this latter category presumably taping the PFC.

These effects on disease progression have implications for our understanding of the dynamics of DA in the PFC and striatum in HD. *COMT* influences DA levels, mostly in the PFC, consistent with the specific effect on cognitive symptoms observed in HD. Indeed, DA antagonists with systemic action, which reduce DA levels in both the PFC and striatum, have been shown to worsen cognitive impairment [27] and chorea intensity at early stages. As in healthy individuals [22], the higher availability of DA in the Met/Met genotype is associated with a preservation of cognitive function at early stages. The greater availability of DA in Met/Met individuals appears to have an effect similar to cognitive reserve in the initial stages of the disease. The effects of high DA levels, which are initially beneficial in the early stages of the disease, eventually become detrimental, due to the long-term toxicity of DA in striatal cells [26].

This biphasic pattern over time suggests a symptomatic, rather than neuroprotective effect. Consistently, early and chronic treatment with the D2 antagonist haloperidol decanoate protects against neuronal dysfunction and aggregate formation in a rat model of HD [55]. *COMT* polymorphism also determines the response to entacapone [29] but not to levodopa. Considering the similar trend of evolution in the cognitive, motor and functional domains, we cannot rule out the possibility of the Val allele being neuroprotective *per se*, since Met/Met individuals display greater gray matter degeneration within DA-innervated structures, including the striatum [51].

**Fig 5:**
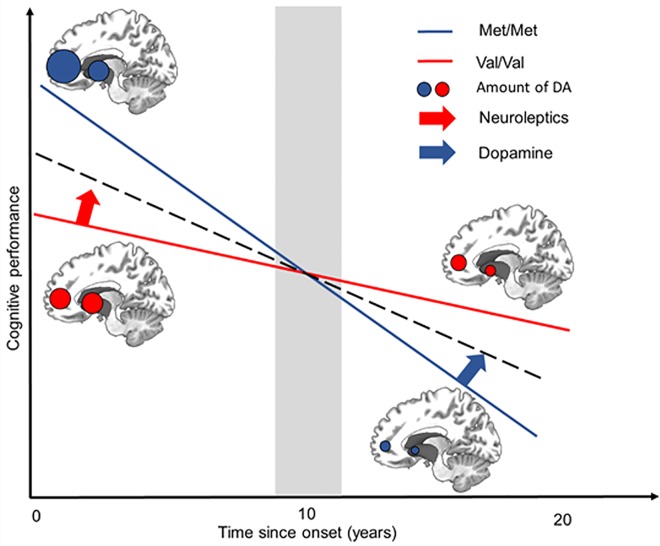
Schematic representation of the biphasic effect of *COMT* polymorphism in HD. In the prefrontal cortex, DA levels are higher in Met/Met HD gene carriers at early stages and in HD gene carriers with premanifest disease than in controls. These levels subsequently decrease over time in both the Met/Met (in blue) and Val/Val groups (in red) [56]. The high levels of DA present in the PFC at early stages result in better cognitive performances. At late stages, higher levels of DA in the PFC in Met/Met HD gene carriers may be toxic, increasing atrophy [26, 51]. In both *COMT* groups, the level of striatal DA decreases over time.

These results open up new possibilities for treatments tailored to patient genotype, slowing disease progression, especially for treatments controlling cognitive function decline, which are currently lacking. It should pave the way for personalized treatment in HD gene carriers by adapting treatment to time- and region-specific changes, taking *COMT* genotype into account. At early stages of the disease, the combination of treatments decreasing DA levels in the striatum and *COMT* inhibitors increasing DA levels in the PFC, might prevent the exacerbation of cognitive deficits, or even improve cognitive ability (Fig 5) in Val/Val HD gene carriers. It has an immediate application in pharmacological management of HD, as inhibitors or activators of *COMT* are already available. At later stages, more than 10 years after onset, it may be harder to target DA levels in the PFC specifically, as classical antipsychotic drugs occupy a large proportion of subcortical dopamine D2 receptors, whereas atypical antipsychotics preferentially occupy cortical 5-HT(2) receptors.

Our study also has practical implications for future clinical trials assessing decline in HD because *COMT* polymorphism appears as an important factor of stratification. Moreover, the methodology we used could be adapted to other neurodegenerative diseases.

## Supporting Information

S1 Fig Distribution of CAG repeats length in the database. (TIF)

S2 Fig Repartition of stages in time according to *COMT* polymorphisms. (TIF)

S3 Fig Link functions between performances at each task and latent processes modelling the domains. We plotted the link function between each task and latent domains. UHDRS motor score **(A)**; UHDRS behavioral **(B)**, IS: Independence Score **(C)**; FAS: Functional Assessment Scale **(D)**, cognitive (letter fluency 1’: at 1 minute **(E)**; letter fluency 2’: at 2 minutes **(F)**; SDMT: symbol digit modalities test **(G)**; Stroop C: Stroop color **(H)**; Stroop W: Stroop word **(I)**; Stroop W/C: Stroop interference **(J**).
(TIF)

S1 Table Demographic characteristics and performance of HD gene carriers including in the longitudinal analysis (N = 350). HD: Huntington’s disease; BMI: body mass index; CAG repeats refers to the number of CAGs in the mutated (*mHtt*) and non-mutated (normal *Htt*) alleles of the Huntingtin gene; UHDRS: Unified Huntington’s Disease Rating Scale; TMS: Total Motor Score; FAS: Functional Assessment Scale; IS: Independence Scale; TFC: Total Functional Capacity; Letter fluency (L Fluency) was tested with PRV letters (French norms) at 1 minute (1’) and 2 minutes (2’); Stroop C: Color; W: Word; W/C: Word/Color (interference score); SDMT: Symbol Digit Modalities Test. Quantitative variables are presented as means, with the standard deviation in brackets, and qualitative variables are presented as frequency counts. Medication use is expressed as a percentage.*Non corrected *P*-values; Chi-squared test for qualitative variables and one-way ANOVA for quantitative data; Ns: not significant.
(DOCX)

S2 Table Modelling results of the sensitivity analysis excluding outliers. The motor domain was modeled including the performances in the TMS; the behavioral domain was modeled including the performances at behavior task of the UHDRS; the functional domain was modeled including the performances at FAS and IS (TFC could not be included because there are not enough values for the model to converge); the cognitive domain was modeled including performances at letter fluency assessed at 1 and 2 minutes, SDMT and the three parts of the Stroop. N: Number of HD gene carriers who have contributed to the estimation (cognitive tasks were not available for all HD gene carriers); SE: Standard error of the estimate, *P*: *P*-values (*** P<0.001, ** P<0.01, *P<0.05). *Baseline* values correspond to the impact of covariates at estimated age at onset. *Slope* values correspond to the impact of covariates on the slope of the decline.
(DOCX)

S3 Table Modelling results of linear mixed models for each tasks. TMS: Total motor score, IS: Independence Scale, FAS: Functional Assessment Scale, SDMT: Symbol Digit Modalities Test, Stroop C: Stroop Color, Stroop W: Stroop Word, Stroop W/C: Stroop interference. N: Number of HD gene carriers who have contributed to the estimation (cognitive tasks were not available for all HD gene carriers); SE: Standard error of the estimate, *P*: *P*-values (*** P<0.001, ** P<0.01, *P<0.05). *Baseline* values correspond to the impact of covariates at estimated age at onset. *Slope* values correspond to the impact of covariates on the slope of the decline.
(DOCX)

## Abbreviations

CAG: Cytosine-Adenine-Guanine
*COMT*: *Catechol-O-Methyltransferase*
DA: Dopamine
FAS: Functional Assessment Scale
HD: Huntington’s disease
Htt: Huntingtin
ICC: Intraclass Correlation Coefficient
IS: Independence Scale
Met: Methionine
mHtt: Mutant Huntingtin
PFC: Prefrontal Cortex
SD: Standard Deviation
SDMT: Symbol Digit Modalities Score
SE: Standard Error
TFC: Total Function Capacity
UHDRS: Unified Huntington’s Disease Rating Scale
Val: Valine
