# Supplementary figures and images for: COMT Val158Met Polymorphism Modulates Huntington's Disease Progression
Source: PLoS One. 2016 Sep 22;11(9):e0161106. doi: 10.1371/journal.pone.0161106 (PMC5033325; doi:10.1371/journal.pone.0161106)

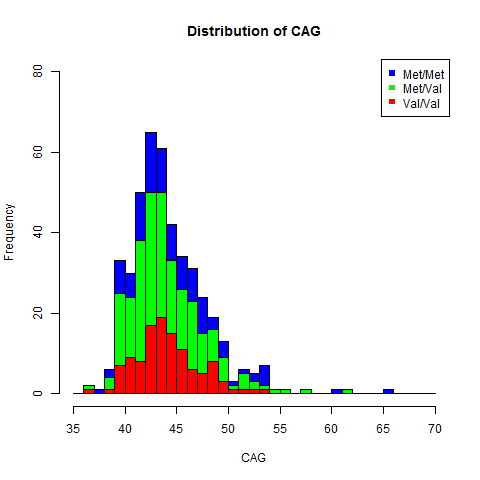

Supplement: S1 Fig — (TIF) [file pone.0161106.s001.tif]

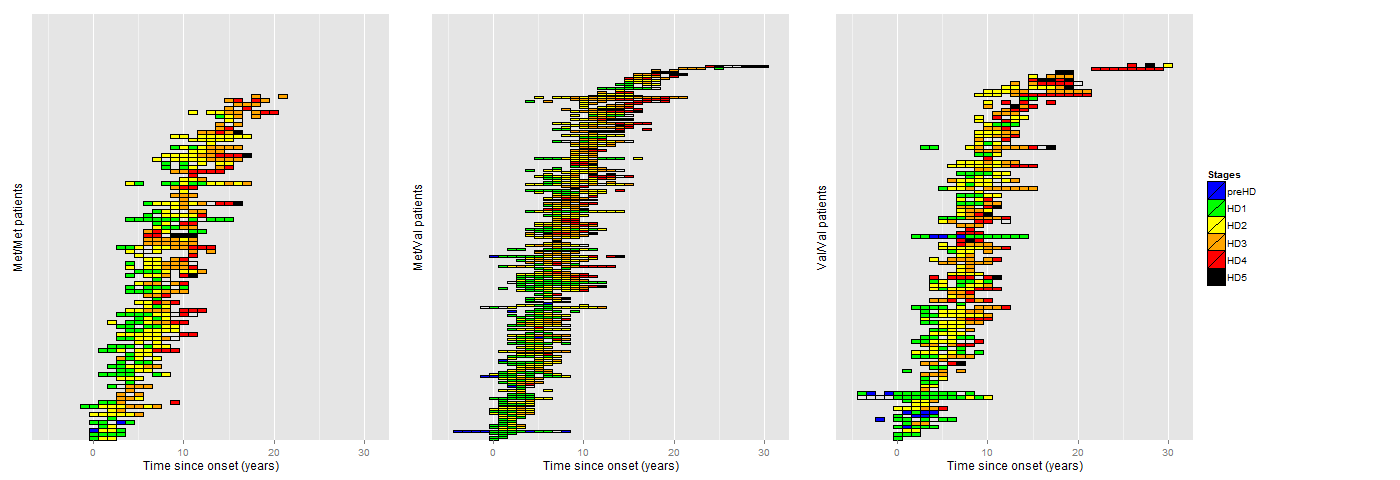

Supplement: S2 Fig — (TIF) [file pone.0161106.s002.tif]

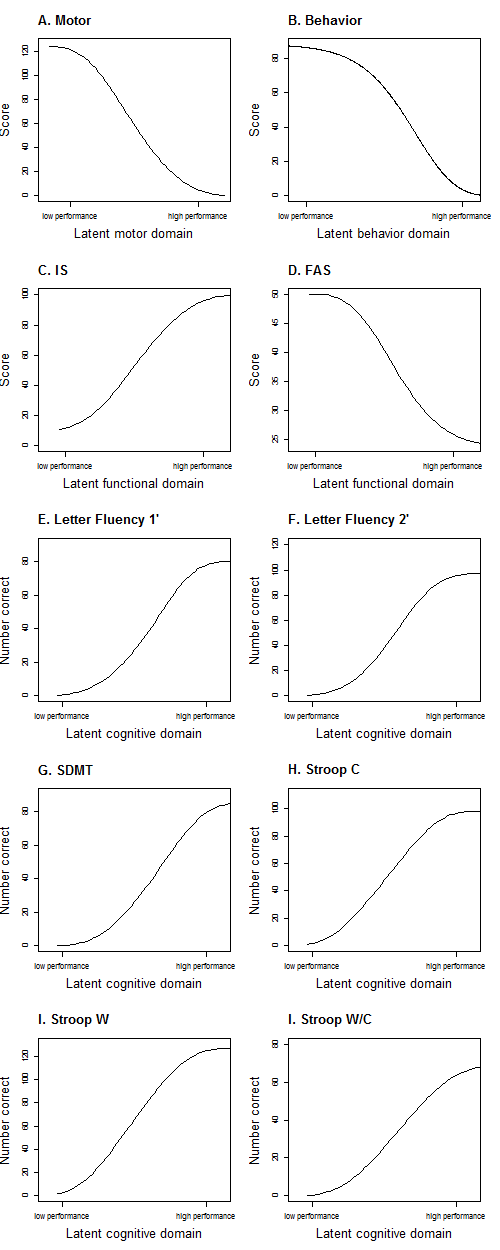

Supplement: S3 Fig — We plotted the link function between each task and latent domains. UHDRS motor score (A); UHDRS behavioral (B), IS: Independence Score (C); FAS: Functional Assessment Scale (D), cognitive (letter fluency 1’: at 1 minute (E); letter fluency 2’: at 2 minutes (F); SDMT: symbol digit modalities test (G); Stroop C: Stroop color (H); Stroop W: Stroop word (I); Stroop W/C: Stroop interference (J). (TIF) [file pone.0161106.s003.tif]
