# Supplementary material for: COMT Val158Met Polymorphism Modulates Huntington's Disease Progression
Source: PLoS One. 2016 Sep 22;11(9):e0161106. doi: 10.1371/journal.pone.0161106 (PMC5033325; doi:10.1371/journal.pone.0161106)
Supplement: S1 Table — HD: Huntington’s disease; BMI: body mass index; CAG repeats refers to the number of CAGs in the mutated (mHtt) and non-mutated (normal Htt) alleles of the Huntingtin gene; UHDRS: Unified Huntington’s Disease Rating Scale; TMS: Total Motor Score; FAS: Functional Assessment Scale; IS: Independence Scale; TFC: Total Functional Capacity; Letter fluency (L Fluency) was tested with PRV letters (French norms) at 1 minute (1’) and 2 minutes (2’); Stroop C: Color; W: Word; W/C: Word/Color (interference score); SDMT: Symbol Digit Modalities Test. Quantitative variables are presented as means, with the standard deviation in brackets, and qualitative variables are presented as frequency counts. Medication use is expressed as a percentage.*Non corrected P-values; Chi-squared test for qualitative variables and one-way ANOVA for quantitative data; Ns: not significant. (DOCX) [file pone.0161106.s004.docx]

**S1 Table. Demographic characteristics and performance of HD gene carriers including in the longitudinal analysis (N=350).**

|  | Met / Met | Met / Val | Val / Val | *p-value** |
| --- | --- | --- | --- | --- |
|  | N=79 | N=175 | N=96 |  |
| Age (yrs) | 47.49 (12.8) | 50.6 (11.6) | 49.6 (10.4) | Ns |
| Sex (% men) | 60.8 | 48.6 | 53.1 | Ns |
| Age at onset (yrs) | 41.6 (11.1) | 45.2 (11.3) | 43.6 (9.7) | Ns |
| Educational level (yrs in education) | 12.2 (3.4) | 11.1 (2.8) | 12.5 (3.5) | 0.0010 |
| BMI | 22.8 (4.0) | 22.7 (3.5) | 22.3 (3.6) | Ns |
| CAG repeats *mHtt* | 45.4 (4.8) | 44.3 (3.6) | 44.5 (3.2) | Ns |
| CAG repeats *Htt* | 18.0 (2.6) | 18.9 (4.1) | 18.9 (4.1) | Ns |
| **UHDRS** |  |  |  |  |
| Motor | 34.8 (17.3) | 34.3 (20.7) | 38.2 (21.7) | Ns |
| Behavior | 19.3 (14.3) | 17.8 (10.8) | 16.9 (12.2) | Ns |
| FAS | 29.7 (4.8) | 29.9 (5.5) | 31.0 (6.0) | Ns |
| IS | 82.7 (13.5) | 82.4 (15.8) | 79.9 (16.6) | Ns |
| TFC | 9.0 (3.2) | 9.3 (3.2) | 8.7 (3.5) | Ns |
| L Fluency 1’ | 20.2 (11.1) | 19.2 (11.4) | 17.2 (11.9) | Ns |
| L Fluency 2’ | 28.9 (17.4) | 27.5 (17.7) | 24.0 (18.2) | Ns |
| Stroop W | 58.1 (21.2) | 60.5 (22.1) | 60.8 (26.8) | Ns |
| Stroop C | 42.3 (16.4) | 42.2 (15.5) | 42.7 (18.7) | Ns |
| Stroop W/C | 21.3 (10.0) | 22.6 (11.7) | 20.2 (13.4) | Ns |
| SDMT | 20.7 (10.4) | 22.2 (13.7) | 21.2 (14.2) | Ns |

HD: Huntington’s disease; BMI: body mass index; CAG repeats refers to the number of CAGs in the mutated (*mHtt*) and non-mutated (normal *Htt*) alleles of the Huntingtin gene; UHDRS: Unified Huntington’s Disease Rating Scale; TMS: Total Motor Score; FAS: Functional Assessment Scale; IS: Independence Scale; TFC: Total Functional Capacity; Letter fluency (L Fluency) was tested with PRV letters (French norms) at 1 minute (1’) and 2 minutes (2’); Stroop C: Color; W: Word; W/C: Word/Color (interference score); SDMT: Symbol Digit Modalities Test. Quantitative variables are presented as means, with the standard deviation in brackets, and qualitative variables are presented as frequency counts. Medication use is expressed as a percentage.*Non corrected *P*-values; Chi-squared test for qualitative variables and one-way ANOVA for quantitative data; Ns: not significant.
