# Supplementary material for: COMT Val158Met Polymorphism Modulates Huntington's Disease Progression
Source: PLoS One. 2016 Sep 22;11(9):e0161106. doi: 10.1371/journal.pone.0161106 (PMC5033325; doi:10.1371/journal.pone.0161106)
Supplement: S2 Table — The motor domain was modeled including the performances in the TMS; the behavioral domain was modeled including the performances at behavior task of the UHDRS; the functional domain was modeled including the performances at FAS and IS (TFC could not be included because there are not enough values for the model to converge); the cognitive domain was modeled including performances at letter fluency assessed at 1 and 2 minutes, SDMT and the three parts of the Stroop. N: Number of HD gene carriers who have contributed to the estimation (cognitive tasks were not available for all HD gene carriers); SE: Standard error of the estimate, P: P-values (*** P<0.001, ** P<0.01, *P<0.05). Baseline values correspond to the impact of covariates at estimated age at onset. Slope values correspond to the impact of covariates on the slope of the decline. (DOCX) [file pone.0161106.s005.docx]

**S2 Table. Modelling results of the sensitivity analysis excluding outliers**

| Domains |  | Motor | (N=312) |  | Behavior | (N=312) |  | Functional | (N=312) |  | Cognitive | (N=308) |
| --- | --- | --- | --- | --- | --- | --- | --- | --- | --- | --- | --- | --- |
|  |  | Estimate | *P* |  | Estimate | *P* |  | Estimate | *P* |  | Estimate | *P* |
|  |  | (SE) | (corrected  *P*) |  | (SE) | (corrected  *P* ) |  | (SE) | (corrected  *P* ) |  | (SE) | (corrected  *P* ) |
| **Baseline:** |  |  |  |  |  |  |  |  |  |  |  |  |
| Met/Val vs Met/Met |  | 0.22 | 0.2285 |  | -0.32 | 0.3603 |  | 0.05 | 0.8227 |  | -0.15 | 0.3669 |
|  |  | (0.19) | (ns) |  | (0.35) | (ns) |  | (0.21) | (ns) |  | (0.17) | (ns) |
| Val/Val vs Met/Met |  | -0.17 | 0.4086 |  | -0.10 | 0.7726 |  | -0.08 | 0.7065 |  | -0.26 | 0.1645 |
|  |  | (0.21) | (ns) |  | (0.34) | (ns) |  | (0.21) | (ns) |  | (0.19) | (ns) |
| Val/Val vs Met/Val |  | -0.40 | 0.0206* |  | 0.22 | 0.3881 |  | -0.13 | 0.4468 |  | -0.11 | 0.4952 |
|  |  | (0.17) | (ns) |  | (0.25) | (ns) |  | (0.17) | (ns) |  | (0.16) | (ns) |
| Number of CAG repeats |  | 0.01 | 0.6999 |  | 0.07 | 0.6226 |  | 0.09 | 0.2328 |  | 0.03 | 0.2001 |
|  |  | (0.03) | (ns) |  | (0.14) | (ns) |  | (0.08) | (ns) |  | (0.03) | (ns) |
| Education level |  | 0.04 | 0.0205 |  | 0.05 | 0.0171* |  | 0.07 | 0.0012** |  | 0.09 | <0.0001*** |
|  |  | (0.02) | (ns) |  | (0.02) | (ns) |  | (0.02) | (0.0048**) |  | (0.02) | (0.0001***) |
| Gender M vs F |  | -0.08 | 0.5970 |  | 0.58 | 0.0126* |  | 0.31 | 0.0372* |  | -0.16 | 0.2125 |
|  |  | (0.15) | (ns) |  | (0.23) | (ns) |  | (0.15) | (ns) |  | (0.13) | (ns) |
| **Slope:** |  |  |  |  |  |  |  |  |  |  |  |  |
| Met/Val vs Met/Met |  | -0.02 | 0.0888 |  | 0.06 | 0.0360* |  | -0.004 | 0.74542 |  | 0.02 | 0.0111* |
|  |  | (0.01) | (ns) |  | (0.03) | (ns) |  | (0.01) | (ns) |  | (0.01) | (ns) |
| Val/Val vs Met/Met |  | 0.02 | 0.2419 |  | 0.04 | 0.2570 |  | -0.005 | 0.7190 |  | 0.02 | 0.0223* |
|  |  | (0.02) | (ns) |  | (0.03) | (ns) |  | (0.01) | (ns) |  | (0.01) | (ns) |
| Val/Val vs Met/Val |  | 0.04 | 0.0012** |  | -0.03 | 0.2694 |  | -0.001 | 0.9427 |  | -0.001 | 0.9357 |
|  |  | (0.01) | (0.0072**) |  | (0.03) | (ns) |  | (0.01) | (ns) |  | (0.01) | (ns) |
| Number of CAG repeats |  | -0.02 | <0.0001*** |  | -0.01 | 0.6056 |  | -0.02 | <0.0001*** |  | -0.02 | <0.0001*** |
|  |  | (0.002) | (<0.0001) |  | (0.01) | (ns) |  | (0.004) | (<0.0001***) |  | (0.002) | (<0.0001***) |
| Gender M vs F |  | 0.03 | 0.0021** |  | -0.06 | 0.0056** |  | -0.01 | 0.2144 |  | 0.03 | <0.0001*** |
|  |  | (0.01) | (0.0084**) |  | (0.02) | (0.0224*) |  | (0.01) | (ns) |  | (0.01) | (0.0002***) |

The motor domain was modeled including the performances at TMS; the behavioral domain was modeled including the performances at behavior task of the UHDRS; the functional domain was modeled including the performances at FAS and IS (TFC could not be included because there are not enough values for the model to converge); the cognitive domain was modeled including performances at letter fluency assessed at 1 and 2 minutes, SDMT and the three parts of the Stroop.

N: Number of HD gene carriers who have contributed to the estimation (cognitive tasks were not available for all HD gene carriers); SE: Standard error of the estimate, *P*: *P*-values (*** P<0.001, ** P<0.01, *P<0.05).

*Baseline* values correspond to the impact of covariates at estimated age at onset. *Slope* values correspond to the impact of covariates on the slope of the decline.
