# Supplementary material for: COMT Val158Met Polymorphism Modulates Huntington's Disease Progression
Source: PLoS One. 2016 Sep 22;11(9):e0161106. doi: 10.1371/journal.pone.0161106 (PMC5033325; doi:10.1371/journal.pone.0161106)
Supplement: S3 Table — TMS: Total motor score, IS: Independence Scale, FAS: Functional Assessment Scale, SDMT: Symbol Digit Modalities Test, Stroop C: Stroop Color, Stroop W: Stroop Word, Stroop W/C: Stroop interference. N: Number of HD gene carriers who have contributed to the estimation (cognitive tasks were not available for all HD gene carriers); SE: Standard error of the estimate, P: P-values (*** P<0.001, ** P<0.01, *P<0.05). Baseline values correspond to the impact of covariates at estimated age at onset. Slope values correspond to the impact of covariates on the slope of the decline. (DOCX) [file pone.0161106.s006.docx]

**S3 Table. Modelling results of linear mixed models for each task**

|  |  | Motor | |  | Behavior | |  | Functional | | | | |
| --- | --- | --- | --- | --- | --- | --- | --- | --- | --- | --- | --- | --- |
|  |  | TMS | (N=348) |  | Behavior | (N=348) |  | IS | (N=348) |  | FAS | (N=348) |
|  |  | Estimate | *P* |  | Estimate | *P* |  | Estimate | *P* |  | Estimate | *P* |
|  |  | (SE) | (corrected *P*) |  | (SE) | (corrected *P*) |  | (SE) | (corrected *P*) |  | (SE) | (corrected *P*) |
| **Baseline:** |  |  |  |  |  |  |  |  |  |  |  |  |
| Met/Val vs Met/Met |  | -0.67 | 0.8374 |  | 1.90 | 0.3395 |  | -1.99 | 0.4536 |  | 1.40 | 0.1662 |
|  |  | (3.28) | (ns) |  | (1.99) | (ns) |  | (2.66) | (ns) |  | (1.01) | (ns) |
| Val/Val vs Met/Met |  | 4.39 | 0.2316 |  | 0.77 | 0.7261 |  | -3.12 | 0.2940 |  | 0.97 | 0.3894 |
|  |  | (3.66) | (ns) |  | (2.19) | (ns) |  | (2.97) | (ns) |  | (1.13) | (ns) |
| Val/Val vs Met/Val |  | 5.06 | 0.0971 |  | -1.13 | 0.5303 |  | -0.12 | 0.6469 |  | -0.43 | 0.6445 |
|  |  | (3.04) | (ns) |  | (1.80) | (ns) |  | (2.45) | (ns) |  | (0.93) | (ns) |
| Number of CAG repeats |  | -0.08 | 0.8170 |  | -0.46 | 0.0255* |  | 0.82 | 0.0022** |  | -0.37 | 0.0003*** |
|  |  | (0.33) | (ns) |  | (0.21) | (ns) |  | (0.27) | (0.0088**) |  | (0.10) | (0.0012**) |
| Education level |  | -0.59 | 0.0725 |  | -0.44 | 0.0048** |  | 0.78 | 0.0017** |  | -0.28 | 0.0032** |
|  |  | (0.33) | (ns) |  | (0.16) | (0.0192*) |  | (0.25) | (0.0068**) |  | (0.10) | (0.0128*) |
| Gender Man versus Woman |  | -0.88 | 0.7287 |  | -4.25 | 0.0057** |  | 3.32 | 0.1074 |  | -1.40 | 0.0732 |
|  |  | (2.54) | (ns) |  | (1.53) | (0.0228*) |  | (2.06) | (ns) |  | (0.78) | (ns) |
| **Slope:** |  |  |  |  |  |  |  |  |  |  |  |  |
| Met/Val vs Met/Met |  | 0.14 | 0.5609 |  | -0.37 | 0.0468* |  | 0.27 | 0.2105 |  | -0.16 | 0.0521 |
|  |  | (0.24) | (ns) |  | (0.19) | (ns) |  | (0.22) | (ns) |  | (0.08) | (ns) |
| Val/Val vs Met/Met |  | -0.30 | 0.2715 |  | -0.18 | 0.3900 |  | 0.11 | 0.6445 |  | 0.003 | 0.9697 |
|  |  | (0.27) | (ns) |  | (0.21) | (ns) |  | (0.24) | (ns) |  | (0.09) | (ns) |
| Val/Val vs Met/Val |  | -0.43 | 0.0516 |  | 0.20 | 0.2531 |  | -0.16 | 0.4285 |  | 0.16 | 0.0324* |
|  |  | (0.22) | (ns) |  | (0.17) | (ns) |  | (0.20) | (ns) |  | (0.07) | (ns) |
| Number of CAG repeats |  | 0.18 | <0.0001*** |  | 0.04 | 0.0494* |  | -0.17 | <0.0001*** |  | 0.07 | <0.0001*** |
|  |  | (0.02) | (<0.0001***) |  | (0.02) | (ns) |  | (0.02) | (<0.0001***) |  | (0.01) | (<0.0001***) |
| Gender Man versus Woman |  | -0.44 | 0.0183 |  | 0.41 | 0.0050** |  | 0.03 | 0.8398 |  | -0.005 | 0.9410 |
|  |  | (0.19) | (ns) |  | (0.15) | (0.0200*) |  | (0.17) | (ns) |  | (0.06) | (ns) |

**(S3 Table continued)**

|  |  | Cognitive | | | | | | | | | | | | | | | | |
| --- | --- | --- | --- | --- | --- | --- | --- | --- | --- | --- | --- | --- | --- | --- | --- | --- | --- | --- |
|  |  | Letter Fluency 1’ | (N=338) |  | Letter Fluency 2’ | (N=339) |  | SDMT | (N=321) |  | Stroop C | (N=329) |  | Stroop W | (N=328) |  | Stroop C/W | (N=325) |
|  |  | Estimate | *P* |  | Estimate | *P* |  | Estimate | *P* |  | Estimate | *P* |  | Estimate | *P* |  | Estimate | *P* |
|  |  | (SE) | (corrected *P*) |  | (SE) | (corrected *P*) |  | (SE) | (corrected *P*) |  | (SE) | (corrected *P*) |  | (SE) | (corrected *P*) |  | (SE) | (corrected *P*) |
| **Baseline:** |  |  |  |  |  |  |  |  |  |  |  |  |  |  |  |  |  |  |
| Met/Val vs Met/Met |  | -3.54 | 0.0941 |  | -6.12 | 0.0424* |  | -0.36 | 0.8647 |  | -3.24 | 0.2365 |  | -2.11 | 0.5667 |  | -0.09 | 0.9625 |
|  |  | (2.11) | (ns) |  | (3.00) | (ns) |  | (2.09) | (ns) |  | (2.73) | (ns) |  | (3.68) | (ns) |  | (1.88) | (ns) |
| Val/Val vs Met/Met |  | -5.69 | 0.0148* |  | -9.70 | 0.0036** |  | -0.18 | 0.9387 |  | -3.50 | 0.2519 |  | -4.00 | 0.3316 |  | -3.42 | 0.1049 |
|  |  | (2.32) | (ns) |  | (3.31) | (ns) |  | (2.28) | (ns) |  | (3.05) | (ns) |  | (4.12) | (ns) |  | (2.10) | (ns) |
| Val/Val vs Met/Val |  | -2.15 | 0.2742 |  | -3.58 | 0.2003 |  | 0.18 | 0.9248 |  | -0.26 | 0.9191 |  | -1.89 | 0.5795 |  | -3.33 | 0.0565 |
|  |  | (1.96) | (ns) |  | (2.79) | (ns) |  | (1.91) | (ns) |  | (2.52) | (ns) |  | (3.41) | (ns) |  | (1.74) | (ns) |
| Number of CAG repeats |  | -0.14 | 0.5215 |  | -0.34 | 0.2764 |  | 0.003 | 0.9898 |  | 0.60 | 0.0280* |  | 0.01 | 0.9820 |  | 0.51 | 0.0067** |
|  |  | (0.22) | (ns) |  | (0.32) | (ns) |  | (0.22) | (ns) |  | (0.27) | (ns) |  | (0.36) | (ns) |  | (0.19) | (ns) |
| Education level |  | 0.94 | <0.0001* |  | 1.69 | <0.0001* |  | 0.77 | 0.0002*** |  | 0.83 | 0.0029** |  | 1.25 | 0.0008*** |  | 0.62 | 0.0011** |
|  |  | (0.20) | (<0.0001*) |  | (0.29) | (<0.0001*) |  | (0.20) | (0.0020**) |  | (0.27) | (0.0290*) |  | (0.37) | (0.0080**) |  | (0.19) | (0.0110*) |
| Gender Man versus Woman |  | -0.51 | 0.7568 |  | 1.49 | 0.5249 |  | -0.86 | 0.5955 |  | -2.22 | 0.2951 |  | -3.98 | 0.1648 |  | -1.95 | 0.1821 |
|  |  | (1.64) | (ns) |  | (2.34) | (ns) |  | (1.62) | (ns) |  | (2.12) | (ns) |  | (2.86) | (ns) |  | (1.46) | (ns) |
| **Slope:** |  |  |  |  |  |  |  |  |  |  |  |  |  |  |  |  |  |  |
| Met/Val vs Met/Met |  | 0.46 | 0.0057** |  | 0.82 | 0.0008*** |  | 0.18 | 0.2630 |  | 0.45 | 0.0243* |  | 0.48 | 0.0805 |  | 0.15 | 0.3069 |
|  |  | (0.17) | (ns) |  | (0.24) | (0.0120*) |  | (0.16) | (ns) |  | (0.20) | (ns) |  | (0.28) | (ns) |  | (0.14) | (ns) |
| Val/Val vs Met/Met |  | 0.56 | 0.0022** |  | 0.87 | 0.0017** |  | -0.15 | 0.3956 |  | 0.23 | 0.3156 |  | 0.50 | 0.1086 |  | 0.40 | 0.0136* |
|  |  | (0.18) | (0.0330*) |  | (0.28) | (0.0255*) |  | (0.17) | (ns) |  | (0.23) | (ns) |  | (0.31) | (ns) |  | (0.16) | (ns) |
| Val/Val vs Met/Val |  | 0.10 | 0.5240 |  | 0.05 | 0.8406 |  | -0.33 | 0.0281* |  | -0.22 | 0.2373 |  | 0.02 | 0.9352 |  | 0.25 | 0.0581 |
|  |  | (0.16) | (ns) |  | (0.24) | (ns) |  | (0.15) | (ns) |  | (0.19) | (ns) |  | (0.26) | (ns) |  | (0.13) | (ns) |
| Number of CAG repeats |  | -0.06 | 0.0015** |  | -0.06 | 0.0146* |  | -0.04 | 0.0117* |  | -0.13 | <0.0001*** |  | -0.12 | <0.0001*** |  | -0.05 | 0.0002*** |
|  |  | (0.02) | (0.0150*) |  | (0.03) | (ns) |  | (0.02) | (ns) |  | (0.02) | (<0.0001***) |  | (0.03) | (<0.0001***) |  | (0.01) | (0.0020**) |
| Gender Man versus Woman |  | 0.06 | 0.6687 |  | -0.10 | 0.5971 |  | 0.16 | 0.2055 |  | 0.38 | 0.0158* |  | 0.78 | 0.0003*** |  | 0.31 | 0.0060** |
|  |  | (0.13) | (ns) |  | (0.20) | (ns) |  | (0.13) | (ns) |  | (0.16) | (ns) |  | (0.22) | (0.0030**) |  | (0.11) | (ns) |

TMS: Total motor score, IS: Independence Scale, FAS: Functional Assessment Scale, SDMT: Symbol Digit Modalities Test, Stroop C: Stroop Color, Stroop W: Stroop Word, Stroop W/C: Stroop interference.

N: Number of HD gene carriers who have contributed to the estimation (cognitive tasks were not available for all HD gene carriers); SE: Standard error of the estimate, *P*: *P*-values (*** P<0.001, ** P<0.01, *P<0.05).

*Baseline* values correspond to the impact of covariates at estimated age at onset. *Slope* values correspond to the impact of covariates on the slope of the decline.
